# Prevalence and correlates of abdominal obesity among adults in Uganda: findings from a national cross-sectional, population based survey 2014

**DOI:** 10.1186/s40608-018-0217-1

**Published:** 2018-12-03

**Authors:** Steven Ndugwa Kabwama, Barbara Kirunda, Gerald Mutungi, Ronald Wesonga, Silver K. Bahendeka, David Guwatudde

**Affiliations:** 1 Makerere University School of Public Health, Kampala, Uganda; 2 Control of Non-Communicable Diseases Desk, Ministry of Health, Kampala, Uganda; 3 School of Statistics and Planning, Makerere University College of Business and Management Sciences, Kampala, Uganda; 4 St. Francis Hospital, Nsambya, Kampala, Uganda; 5 Department of Epidemiology & Biostatistics, School of Public Health, Makerere University College of Health Sciences, Kampala, Uganda

**Keywords:** Abdominal obesity, Non-communicable diseases, WHO STEPs methodology, Sub-Saharan Africa, Uganda

## Abstract

**Background:**

Overweight and obesity are associated with health complications the gravity of which, vary with the regional deposition of the excess fat. The Body Mass Index (BMI) is often used to measure obesity although is an inferior predictor of cardiovascular disease risk mortality and morbidity compared with measures of abdominal obesity. We analyzed data from Uganda’s 2014 World Health Organization (WHO) STEPwise approach to surveillance of Non-communicable diseases (NCDs) survey to estimate the prevalence of abdominal obesity and associated factors to provide information on the prevention and control of overweight and obesity.

**Methods:**

Data were collected using the WHO STEPS protocol. Waist measurement was taken using a non-stretchable standard tape measure mid-way between the lowest rib and iliac crest with the subject standing at the end of gentle expiration. Participants with waist circumference > 102 cm for men and 88 cm for women were classified as abdominally obese. We used weighted modified Poisson regression with robust error variance to estimate the prevalence of abdominal obesity and associated factors.

**Results:**

Of the 3676 participants, 432 (11.8%) were abdominally obese; with the prevalence higher among females 412 (19.5%) compared with males 20 (1.3%). Compared with males, female participants were more likely to be abdominally obese Adjusted Prevalence Rate Ratio (APRR) 7.59 [5.58–10.33]. Participants who were married or cohabiting APRR 1.82 [1.29–2.57] and participants who were separated or divorced APRR 1.69 [1.17–2.46] were more likely to be abdominally obese compared with those who had never married before. Compared with rural dwellers, participants from urban areas were more likely to be abdominally obese APRR 1.29 [1.09–1.53]. Compared with participants with normal blood pressure, those with elevated blood pressure were more likely to be abdominally obese APRR 1.83 [1.57–2.14].Compared with participants without any education, those with secondary education were more likely to be abdominally obese APRR 1.42 [1.12–1.78].

**Conclusions:**

There is a high prevalence of abdominal obesity among adults in Uganda which puts many at risk of developing associated metabolic complications. These data provide useful information for developing interventions and formulation of policies for the control and prevention of abdominal obesity in Uganda.

## Background

The significance of obesity as a global public health challenge cannot be over emphasized. Cross country comparisons have shown steady increases in the prevalence of overweight and obesity in Africa which are envisaged to reach epidemic proportions in the near future if left unabated [1]. Between 1995 and 2011 the prevalence of overweight and obesity in Uganda increased more than 2-fold from 8 to 18.8% according to the Uganda Demographic Health Surveys (UDHS) [2, 3]. Overweight and obesity are associated with complications that include hypertension, atherosclerosis, diabetes and many types of cancers [4]. Aside from the degree of obesity, the regional distribution of fat is an important determinant of the health hazards associated with obesity. For example increased intra-abdominal fat has been associated with increased glucose intolerance [5], mortality from all causes, cardiovascular diseases and cancers [6]. The Body Mass Index (BMI) (kg/m^2^) is commonly used to quantify obesity and then classify individuals as underweight, normal weight, overweight or different subgroups of obesity. Although BMI has been shown to correlate well with measures of abdominal obesity at the population level, persons with higher readings of abdominal obesity will have more abdominal fat at any given BMI value at the individual level [7]. It has been shown that after adjusting for BMI, the risk of cardiovascular disease mortality is higher among persons with higher waist-circumference [6]. In fact, a meta analysis has shown that measures of abdominal obesity are superior to BMI in the detection of risk factors for cardiovascular diseases in both women and men [8].

Previously, there have not been any national population studies on obesity in Uganda. Some studies however have been conducted among young adults [9], adolescents [10], women and children [11], and some in selected districts representing urban and peri-urban settings [12]. Furthermore, all these studies used BMI as an indicator of obesity and therefore could not distinguish abdominal obesity from overall obesity.

In 2014, a nationwide survey was carried out in Uganda to provide baseline estimates of the major risk factors for Non-Communicable Diseases (NCDs). We analyzed data from this survey to estimate the prevalence of abdominal obesity and identify the associated factors to provide information for the prevention and control of abdominal obesity and its associated complications among adults in Uganda.

## Methods

### Study design

The Uganda national NCD risk factor survey was conducted using a cross-sectional study design. Data were collected using the World Health Organization’s (WHO) STEPwise approach to surveillance, a global standardized protocol for assessment of risk factors for NCDs [13]. A detailed description of the conduct, sampling procedures and methods used to conduct the survey has been described elsewhere [14–16]. Here we only describe the methods relevant to the content of this paper.

### Measurements

Briefly, in STEP 1, the survey collected data on socio-demographic and behavioral characteristics including alcohol use, tobacco use, fruit and vegetable intake and level of physical activity (PA).

In STEP 2, physical measurements were made including weight, height, blood pressure, waist circumference and hip circumference. Three blood pressure readings were taken and the average of the last two was used in analysis. A participant was classified as being hypertensive if their average systolic blood pressure was ≥140 mmHg, and/or their average diastolic blood pressure was ≥90 mmHg [17] or if they reported being on any medication for raised blood pressure.

Waist and hip circumferences were measured to the nearest 0.5 of a centimeter using a non-stretchable standard tape measure. The waist measurement was taken mid-way between the lowest rib and the iliac crest with the subject standing at the end of gentle expiration, and hip measurement at the greater trochanters [18]. Participants were ineligible for the waist and hip measurement if pregnant, chair-bound or had a colostomy/ileostomy.

Before the waist and hip circumferences were measured, the Research Assistant requested the participant to wear light clothing. An explanation about the importance of the measurements and that clothing can substantially affect the circumference reading were provided to the participant. Thus where found necessary participants were asked to remove all outer layers of clothing, such as jackets, heavy or baggy jumpers, cardigans and waistcoats, shoes with high heels, tight garments intended to alter the shape of the body, such as corsets, lycra body suits and support tights. If the participant was wearing a belt, they were requested to remove it or loosen it for the measurements. Research Assistants received training on how to conduct these measurements during a five-day training prior to initiation of the survey.

In STEP 3, a finger-prick blood sample was collected to assess the level of plasma glucose (FPG) in the fasting state. Participants with a FPG of at least 6.1 to 6.9 mmol/L were classified as pre-diabetic while those with at least 7.0 mmol/L or on treatment for diabetes mellitus were classified as diabetic [19].

### Statistical analysis

Based on an expert consultation report of 2008, the WHO provided cut-off points of waist circumference and waist-hip ratios, that suggest increased risk for metabolic complications [20, 21]. A waist circumference > 94 cm for men and > 80 cm for women indicates increased risk for metabolic complications and a waist circumference > 102 cm for men and > 88 cm for women indicates substantially increased risk for metabolic complications and a need for weight management [22]. In the analysis for this article, participants at a substantially increased risk for metabolic complications were described as abdominally obese [22, 23]. Waist circumference was preferred over waist-hip ratio as an indicator of abdominal obesity because a preliminary analysis revealed that the two measures were positively correlated. In addition, a comparative evaluation of waist-circumference, waist-hip ratio and BMI showed that waist circumference was superior to both waist-hip ratio and BMI in the prediction of single and multiple cardiovascular disease risk factors [24]. The prevalence of abdominal obesity was calculated as the percentage of participants with higher than the above stated cut-off measurements. We used the Pearson’s Chi-square statistic to assess whether there was an association between the prevalence of abdominal obesity among males and females and the various participant characteristics. The Pearson’s Chi-square statistic or the Chi-square test for independence assesses whether two categorical variables are associated/ related. In our analysis, the null hypothesis (H_0_) was that there was no association between the prevalence of abdominal obesity among males and females and any of the participant characteristics i.e. that the prevalence of abdominal obesity among males and females was the same across the participant sub-categories. The null hypothesis was rejected at a *p*-value of < 0.05.

To identify factors associated with abdominal obesity, we used weighted modified Poisson regression with robust variance [25], to estimate both the crude and adjusted prevalence rate ratios (PRR), with their corresponding 95% confidence intervals. Modified Poisson regression with robust error variance was used because there is a tendency to over-estimate the prevalence and under-estimate the standard errors of the estimated risk-ratios when the outcome is not rare as is the case with logistic regression [26]. To account for differing sampling proportions of participants per enumeration area, all analyses were done using sampling selection weights. All variables suspected to be associated with abdominal obesity were fitted into a model and removed one at time starting with the variable with the highest *p*-value until only those with a *p*-value < 0.05 were left in the model. The independent variables included in the model were age, sex, and marital status, level of education, geographical region of residence, urban or rural residence, employment status, fruit and vegetable consumption, level of physical activity, alcohol use, blood pressure and FPG. All statistical analyses were performed using STATA version 12 (StataCorp, College Station, Texas, USA).

## Results

### Participants and their characteristics

From the sample size calculation, 4900 participants were targeted of whom 3987 agreed to participate, yielding a participation rate of 81.4%. Of the 3987 participants, 3676 had measurements for both waist and hip circumference and are included in this analysis. Of the 3676 participants, 1562 (42.5%) were males, 2688 (73.1%) resided in rural areas and 2398 (65.3%) were either married or cohabiting (Table 1).

**Table 1:** Characteristics of participants, Uganda STEPS survey 2014

Characteristic	-*n*-	%
Sex		
Male	1562	42.5
Female	2114	57.5
Age group, years		
18–24	846	23.0
25–34	1089	29.6
35–44	795	21.6
45–54	538	14.6
55–64	289	7.9
≥ 65	119	3.2
Mean ± SD	35.8 ± 13.1	
Residence		
Rural	2688	73.1
Urban	988	26.9
Region		
Eastern	1178	32.1
Central	904	24.6
Northern	695	18.9
Western	899	24.5
Ethnicity		
Baganda	713	19.4
Banyankore/Bakiga	667	18.2
Basoga	291	7.9
Banyoro/Batooro	236	6.4
Lugbara/Madi	229	6.2
Other	1539	41.9
Marital status		
Never married	602	16.4
Married/Cohabiting	2398	65.3
Separated/Divorced/Widowed	673	18.3
Completed level of education		
No formal school	612	16.7
Primary school	1497	40.9
Secondary school	1203	32.8
University or higher	351	9.6
Employment status		
Not employed	1257	34.2
Employed	2418	65.8

### Prevalence of abdominal obesity among adults in Uganda

Among the males, 20 (1.3%) were abdominally obese as were 412 (19.5%) among the females, (*p* < 0.05) (Table 2). Overall, 432 (11.8%) were abdominally obese. In urban areas 160 (16.2%) were abdominally obese as were 272 (10.1%) in rural areas. The prevalence of abdominal obesity among men and women was significantly associated with marital status (*p* = 0.01). Men had a mean waist circumference of 77.4 cm while women had a mean waist circumference of 80.7 cm, and 79.3 cm overall.

**Table 2:** Prevalence of abdominal obesity among women and men in Uganda, STEPS survey 2014

Variable	-*n*-	# of persons with abdominal obesity –*n*-(%)	Males *n* = 1562	Females *n* = 2114	p*
All	3676	432 (11.8)	20 (4.6)	412 (95.4)	< 0.05
Age					
18–24	846	41 (4.8)	3 (7.3)	38 (93.7)	0.31
25–34	1089	99 (9.1)	1 (1)	98 (99)	
35–44	795	124 (15.6)	7 (5.6)	117 (94.4)	
45–54	538	91 (16.9)	5 (5.5)	86 (94.5)	
55–64	289	55 (19.0)	4 (7.3)	51 (92.7)	
≥ 65	119	22 (18.5)	0 (0)	22 (100)	
Rural-urban residence					
Urban	988	160 (16.2)	11 (6.9)	149 (93.1)	0.09
Rural	2688	272 (10.1)	9 (3.3)	263 (96.7)	
Marital status					
Never married	602	22 (3.6)	3 (13.6)	19 (86.4)	0.01
Married/Cohabiting	2398	283 (11.8)	16 (5.7)	267 (94.3)	
Other	673	126 (18.7)	1 (0.8)	125 (99.2)	
Employment					
Employed	2418	304 (12.6)	18 (5.9)	286 (94.1)	0.05
Unemployed	1257	128 (10.2)	2 (1)	126 (99.0)	
Fruit & Veg servings/ day					
≥ 5	566	59 (10.4)	3 (5.1)	56 (94.9)	0.93
1–4	2958	353 (11.9)	17 (4.8)	336 (95.2)	
Met WHO recommendation for physical activity					
Yes	3574	413 (11.6)	18 (4.4)	395 (95.6)	0.21
No	102	19 (18.6)	2 (10.5)	17 (89.5)	
Alcohol use in standard drinks per occasion					
Never	1905	253 (13.3)	9 (3.6)	244 (96.4)	0.06
Ever or ≤ 6	1385	145 (10.5)	6 (4.1)	139 (95.9)	
> 6	361	31 (8.6)	4 (12.9)	27 (87.1)	
Hypertensive					
No	2705	241 (8.9)	7 (2.9)	234 (97.1)	0.06
Yes	971	191 (19.7)	13 (6.8)	178 (93.2)	
FPG (mmol/L)^c^					
< 6.1	3309	376 (11.4)	13 (3.5)	363 (96.5)	< 0.05
6.1–6.9	58	9 (15.5)	3 (33.3)	6 (66.7)	
> 7.0 or on DM Rx	309	47 (15.2)	4 (8.5)	43 (91.5)	

^c^Fasting Plasma Glucose in mmol/L

p* *p*-value for a Chi-square test for independence comparing the prevalence of abdominal obesity among males and females across participant sub-categories

### Factors associated with abdominal obesity

The factors significantly associated with abdominal obesity were sex, marital status, level of education, urban/ rural residence and blood pressure. Compared with males, female participants were more likely to be abdominally obese APRR 7.59 [5.58–10.33] (Table 3). Participants who were married or cohabiting APRR 1.82 [1.29–2.57] and participants who were separated or divorced APRR 1.69 [1.17–2.46] were more likely to be abdominally obese compared with participants who had never married before. Compared with rural dwellers, participants from urban areas were more likely to be abdominally obese APRR 1.29 [1.09–1.53]. Compared with participants with normal blood pressure, participants with elevated blood pressure or on treatment for elevated blood pressure were more likely to be abdominally obese APRR 1.83 [1.57–2.14]. Compared with participants without any education, participants with secondary education were more likely to be abdominally obese APRR 1.42 [1.12–1.78].

**Table 3:** Factors associated with abdominal obesity among adults in Uganda, STEPS survey 2014

Variable	Males		Females		Males and Females	
	Crude PRR^a^ [95% CI]	Adjusted PRR [95% CI]*	Crude PRR [95% CI]	Adjusted PRR [95% CI]**	Crude PRR [95% CI]	Adjusted PRR [95% CI]***
Sex						
Males	–	–	–	–	1.0	1.0
Females	–	–	–	–	8.70 [6.19–12.22]	7.59 [5.58–10.33]
Age						
18–24	1.0	1.0	1.0	1.0	1.0	1.0
25–34	0.56 [0.21–1.49]	0.76 [0.29–1.97]	1.04 [0.80–1.34]	0.97 [0.76–1.25]	1.00 [0.78–1.28]	0.97 [0.76–1.25]
35–44	0.35 [0.13–0.99]	1.03 [0.40–2.63]	1.11 [0.85–1.48]	1.08 [0.82–1.42]	1.04 [0.80–1.35]	1.05 [0.81–1.36]
45–54	0.42 [0.12–1.43]	0.64 [0.22–1.86]	0.92 [0.67–1.26]	0.90 [0.66–1.23]	0.85 [0.62–1.16]	0.86 [0.63–1.17]
55–64	0.27 [0.07–1.08]	0.62 [0.20–1.91]	1.08 [0.75–1.57]	1.02 [0.72–1.46]	0.97 [0.68–1.39]	0.94 [0.66–1.33]
≥ 65	0.39 [0.05–2.89]	0.58 [0.12–2.77]	1.23 [0.79–1.91]	1.22 [0.84–1.78]	1.11 [0.71–1.73]	1.11 [0.75–1.65]
Marital status						
Never married	1.0	1.0	1.0	1.0	1.0	1.0
Married/Cohabiting	2.01 [0.90–4.51]	1.16 [0.59–2.27]	2.03 [1.31–3.15]	2.08 [1.39–3.13]	1.85 [1.27–2.70]	1.82 [1.29–2.57]
Other	1.56 [0.52–4.68]	0.68 [0.26–1.82]	1.83 [1.14–2.91]	1.92 [1.25–2.94]	1.67 [1.11–2.50]	1.69 [1.17–2.46]
Level of education						
No formal schooling	1.0	1.0	1.0	1.0	1.0	1.0
Primary	0.57 [0.16–2.03]	0.60 [0.24–1.50]	1.18 [0.95–1.47]	1.18 [0.95–1.46]	1.15 [0.91–1.46]	1.17 [0.94–1.46]
Secondary	1.14 [0.34–3.76	1.08 [0.42–2.81]	1.44 [1.13–1.83]	1.40 [1.12–1.75]	1.42 [1.10–1.84]	1.42 [1.12–1.78]
University or higher	0.71 [0.19–2.68]	0.92 [0.31–2.70]	1.18 [0.82–1.68]	1.12 [0.78–1.60]	1.15 [0.80–1.66]	1.19 [0.84–1.67]
Region of residence						
Eastern	1.0	1.0	1.0	1.0	1.0	1.0
Central	0.64 [0.22–1.85]	0.54 [0.22–1.31]	0.99 [0.76–1.28]	0.93 [0.72–1.20]	0.96 [0.75–1.23]	0.87 [0.69–1.11]
Northern	1.26 [0.54–2.89]	0.85 [0.37–1.98]	1.15 [0.92–1.44]	1.08 [0.86–1.36]	1.13 [0.89–1.44]	1.03 [0.82–1.30]
Western	0.86 [0.35–2.11]	0.51 [0.20–1.31]	1.23 [0.99–1.52]	1.18 [0.96–1.45]	1.19 [0.96–1.48]	1.15 [0.94–1.41]
Rural-urban residence						
Rural	1.0	1.0	1.0	1.0	1.0	1.0
Urban	1.47 [0.68–3.18]	1.80 [0.98–3.31]	1.33 [1.10–1.60]	1.29 [1.09–1.54]	1.31 [1.08–1.58]	1.29 [1.09–1.53]
Employment						
Unemployed	1.0	1.0	1.0	1.0	1.0	1.0
Employed	0.72 [0.33–1.54]	0.93 [0.45–1.91]	1.25 [1.05–1.49]	1.22 [1.04–1.43]	1.22 [1.01–1.47]	1.18 [0.99–1.40]
Fruit & Veg servings/ day						
≥ 5	1.0	1.0	1.0	1.0	1.0	1.0
1–4	0.69 [0.32–1.47]	0.85 [0.38–1.91]	0.92 [0.75–1.13]	0.94 [0.77–1.15]	0.90 [0.73–1.12]	0.93 [0.75–1.15]
Met WHO recommendation for physical activity						
Yes	1.0	1.0	1.0	1.0	1.0	1.0
No	1.76 [0.28–10.92]	1.55 [0.37–6.42]	1.03 [0.66–1.61]	0.92 [0.59–1.45]	1.05 [0.70–1.59]	0.90 [0.59–1.35]
Alcohol use in standard drinks per occasion						
Never	1.0	1.0	1.0	1.0	1.0	1.0
Ever or ≤ 6	1.44 [0.74–2.78]	0.89 [0.45–1.75]	1.00 [0.84–1.19]	0.97 [0.82–1.16]	1.01 [0.85–1.21]	0.99 [0.83–1.17]
> 6	1.53 [0.61–3.85]	1.07 [0.47–2.45]	1.15 [0.81–1.64]	1.12 [0.79–1.59]	1.19 [0.85–1.65]	1.20 [0.87–1.66]
Hypertensive						
No	1.0	1.0	1.0	1.0	1.0	1.0
Yes	8.85 [4.19–18.71]	10.15 [4.78–21.53]	1.59 [1.33–1.99]	1.60 [1.36–1.89]	1.79 [1.52–2.12]	1.83 [1.57–2.14]
FPG (mmol/L)						
< 6.1	1.0	1.0	1.0	1.0	1.0	1.0
6.1–6.9	3.98 [1.14–13.91]	2.52 [0.80–8.00]	0.98 [0.55–1.78]	0.99 [0.56–1.78]	1.18 [0.72–1.95]	1.22 [0.74–2.01]
> 7.0, or on DM Rx	2.24 [1.05–4.76]	2.45 [1.32–4.54]	1.14 [0.88–1.48]	1.13 [0.88–1.45]	1.22 [0.94–1.59]	1.24 [0.96–1.60]

^**a**^Prevalence Rate Ratio

*Adjusted for blood pressure and FPG

**Adjusted for marital status, level of education, urban/rural residence, employment status and blood pressure

***Adjusted for sex, marital status, level of education, urban/rural residence and blood pressure

## Discussion

The survey estimated that 11.8% of adults in Uganda are abdominally obese with men and women having a mean waist circumference of 77.4 cm and 80.7 cm respectively. Sociologically, the high prevalence of abdominal obesity in Uganda has been attributed to a society in which plumpness is associated with wealth, respect, dignity, confidence and beauty [27, 28].

Compared with Uganda, the mean waist circumference reported in Botswana was higher among both men (82.5 cm) and women (88.4 cm) [29]. Similarly, the mean waist circumference reported in Tanzania was higher among both men (80.6 cm) and women (84.9 cm) [30] compared to the results of this study. In Kenya however, although the mean waist circumference was higher (78.6 cm) among men compared with Uganda, the women in Kenya had a lower mean waist circumference (79.1 cm) [31].

All the surveys consistently reported higher mean waist circumference among women compared with men. In the association analysis, sex was also found to be a significant predictor of abdominal obesity. This difference in the extent of abdominal fat deposition between women and men is no surprise finding and has been documented elsewhere [32–34]. It has been attributed to the difference in sex steroid hormones that drive the divergence in body structure and composition particularly during adolescence. In fact, even among men, the amount of free and total testosterone is inversely associated with the degree of abdominal obesity [35]. Some researchers however have further attributed this difference to the dissimilarities in the environment and genetic susceptibility of fat accumulation [36] between men and women. Biologically though, the increased waist circumference in females could also be related to increasing parity [37] as pregnancy leads to an increase in visceral and abdominal fat postpartum [20] and a post-menopausal redistribution of body fat to the abdominal area [38]. Interventions for the control and prevention of unhealthy abdominal weight gain should bear in mind the biological differences between men and women and address the socio-cultural perceptions surrounding abdominal obesity in Uganda.

We also found that participants who were married, separated or divorced were more likely to be abdominally obese. The literature on the association between marital status and abdominal obesity is inconsistent. Some researchers have reported similar positive associations between being married or formerly married and weight gain [39] while others have reported different patterns among married people, between women and men [40]. The likelihood of married people being more abdominally obese has been attributed to a change of dietary patterns after marriage and increased social support. Married people are more likely to have a more stable eating pattern and the social support that comes from the responsibility of eating together [41].

The analysis also revealed a consistent association between high blood pressure and abdominal obesity. Similar associations have been reported in cross-sectional studies [42] and longitudinal studies [43]. After following up participants for 13 years, Larsson and colleagues posited that it was because the adipocytes around the abdomen are unique in such a way that they deposit their free fatty acids directly into the portal vein which increases the amount of fatty acids in the liver. The elevated free fatty acid concentration results in an increase in the contents of the portal blood vessels leading to high blood pressure [44]. Strategies aimed at the control and prevention of abdominal obesity should entail screening to identify persons with elevated blood pressure for early detection, management and treatment.

The analysis also revealed significant associations between urban or rural residence, level of education and abdominal obesity. Urban residence has previously been associated with obesity [11] and has been attributed to the rapid globalization and trends towards unhealthy diets and sedentary lifestyles [45] in urban areas. Higher level of education might explain abdominal obesity because it is a proxy indicator of higher socio-economic status. In Uganda, overweight, raised blood pressure and raised cholesterol are common among people with higher socio-economic status [46] which can also be reflected in the level of education [47]. Higher education and wealth have also been shown to correlate with higher weight status among people in Uganda [3]. Strategies for economic development should also promote active lifestyles and healthy food choices so that the socio-economic gains are not diluted by the individual and health system costs of treating abdominal obesity related complications.

### Strengths and limitations

The findings of this study are subject to a number of limitations. First, because mean waist circumference can depend on ethnic group and gender, and yet the cut-offs for abdominal obesity that were used were based on reports from European populations [20], it is likely that there was misclassification of those at risk. However the few studies that have reported the cut-offs for abdominal obesity and risk of metabolic complications in African populations have quoted more conservative cut-offs [48, 49]. This means that the misclassification led to under-estimation and not over-estimation of the prevalence consequently biasing the associations towards the null. Secondly because of the cross-sectional nature of the study design, we cannot make inferences on causality. Also, information on socio-demographic characteristics and some risk factors were assessed using self-reporting which is a source of information bias. However the main outcome measure was assessed using a standard protocol. In addition, findings from the survey are representative of the Uganda adult population and can be compared with findings from other countries that used the same protocol.

## Conclusions

There is a high prevalence of abdominal obesity among adults in Uganda which puts many at risk of developing associated metabolic complications. These data provide useful information for developing interventions and formulation of policies for the control and prevention of abdominal obesity in Uganda.

## Abbreviations

APRR: Adjusted Prevalence Rate Ratio
BMI: Body Mass Index
NCD: Non Communicable Diseases
PRR: Prevalence Rate Ratio
STEPS: Stepwise Approach to Surveillance
UDHS: Uganda Demographic Health Survey
WHO: World Health Organization

## References

[1] Ziraba AK, Fotso JC, Ochako R (2009). Overweight and obesity in urban Africa: a problem of the rich or the poor?. BMC Public Health.

[2] Uganda Bureau of Statistics (UBOS) and ICF International Inc. Uganda Demographic and Health Survey (2006). Kampala Uganda: UBOS and Claverton.

[3] Uganda Bureau of Statistics (UBOS) and ICF International Inc. Uganda Demographic and Health Survey (2011). Kampala Uganda: UBOS and Claverton.

[4] Bray GA (1985). Complications of obesity. Ann Intern Med.

[5] Despres J (1992). Abdominal obesity as important component of insulin-resistance syndrome. J Nutr.

[6] Zhang C, Rexrode KM, van Dam RM, Li TY, Hu FB (2008). Abdominal obesity and the risk of all-cause, cardiovascular, and cancer mortality sixteen years of follow-up in US women. Circulation.

[7] Després J-P (2012). Body fat distribution and risk of cardiovascular disease an update. Circulation.

[8] Lee Crystal Man Ying, Huxley Rachel R., Wildman Rachel P., Woodward Mark (2008). Indices of abdominal obesity are better discriminators of cardiovascular risk factors than BMI: a meta-analysis. Journal of Clinical Epidemiology.

[9] Baalwa J, Byarugaba B, Kabagambe E, Otim A. Prevalence of overweight and obesity in young adults in Uganda. Afr J Health Sci. 2010;10(4). PMC3052810 21416039

[10] Peltzer K, Pengpid S (2011). Overweight and obesity and associated factors among school-aged adolescents in Ghana and Uganda. Int J Environ Res Public Health.

[11] Turi KN, Christoph MJ, Grigsby-Toussaint DS (2013). Spatial distribution of underweight, overweight and obesity among women and children: results from the 2011 Uganda demographic and health survey. Int J Environ Res Public Health.

[12] Kirunda BE, Fadnes LT, Wamani H, Van den Broeck J, Tylleskär T (2015). Population-based survey of overweight and obesity and the associated factors in peri-urban and rural eastern Uganda. BMC Public Health.

[13] World Health Organization. STEPwise approach to surveillance (STEPS) 2015. Available from: http://www.who.int/chp/steps/en/. Accessed 18 Sep 2015.

[14] Guwatudde D, Mutungi G, Wesonga R, Kajjura R, Kasule H, Muwonge J (2015). The epidemiology of hypertension in Uganda: findings from the National non-Communicable Diseases Risk Factor Survey. PLoS One.

[15] Guwatudde D, Kirunda BE, Wesonga R, Mutungi G, Kajjura R, Kasule H (2016). Physical activity levels among adults in Uganda: findings from a countrywide cross-sectional survey. J Phys Act Health.

[16] Bahendeka S, Wesonga R, Mutungi G, Muwonge J, Neema S, Guwatudde D (2016). Prevalence and correlates of diabetes mellitus in Uganda: a population-based national survey. Tropical Med Int Health.

[17] American Diabetes Association (2014). Standards of medical Care in Diabetes. Diabetes Care.

[18] National Heart Lung and Blood Institute, National Institutes of Health, North American Association for the study of Obesity. The Practical Guide Identification, Evaluation, and Treatment of Overweight and Obesity in Adults. 2000 Contract No.: No. 00–4084. Available from https://www.nhlbi.nih.gov/files/docs/guidelines/prctgd_c.pdf. Accessed 15 Dec 2016.

[19] World Health Organizatin (1999). Diagnosis and Cassification of Diabetes Mellitus. Definition, Diagnossis and Classification of Diabetes Mellitus and its Complications.

[20] World Health Organization. Waist circumference and waist-hip ratio. Report of a WHO Expert Consultation. Geneva, Switzerland; 2008. Available from http://apps.who.int/iris/bitstream/10665/44583/1/9789241501491_eng.pdf. Accessed 23 Nov 2016.

[21] Grundy SM, Cleeman JI, Daniels SR, Donato KA, Eckel RH, Franklin BA (2005). Diagnosis and management of the metabolic syndrome: an American Heart Association/National Heart, Lung, and Blood Institute scientific statement. Circulation.

[22] Lean M, Han T, Morrison C (1995). Waist circumference as a measure for indicating need for weight management. BMJ.

[23] Lamarche B (1998). Abdominal obesity and its metabolic complications: implications for the risk of ischaemic heart disease. Coron Artery Dis.

[24] Dobbelsteyn C, Joffres M, MacLean DR, Flowerdew G (2001). A comparative evaluation of waist circumference, waist-to-hip ratio and body mass index as indicators of cardiovascular risk factors. The Canadian heart health surveys. Int J Obes Rela Metab Disord.

[25] Zou G (2004). A modified poisson regression approach to prospective studies with binary data. Am J Epidemiol.

[26] Yelland LN, Salter AB, Ryan P (2011). Performance of the modified Poisson regression approach for estimating relative risks from clustered prospective data. Am J Epidemiol.

[27] Puoane T, Fourie J, Shapiro M, Rosling L, Tshaka N, Oelefse A (2005). ‘Big is beautiful’–an exploration with urban black community health workers in a south African township. South Afr J Clin Nutr.

[28] Janzon E, Namusaazi S, Bolmsjö I. Increasing obesity in Ugandan women due to transition from rural to urban living conditions? A qualitative study on traditional body image, changed lifestyles and unawareness of risk for heart disease. J Res Obes. 2015. 10.5171/2015.213083.

[29] Ministry of Health Republic of Botswana, World Health Organization. Republic of Botswana Chronic Disease Risk Factor Surveillance Report 2007. 2007. Available from http://www.who.int/chp/steps/2007_STEPS_Report_Botswana.pdf. Accessed 7 Sep 2015.

[30] Mayige M. Tanzania STEPS Survey 2012 Fact Sheet. 2012. Available from http://www.who.int/chp/steps/UR_Tanzania_FactSheet_2012.pdf?ua=1. Accessed 7 Sep 2015.

[31] Kenya Ministry of Health Division of Non Communicable Diseases, Kenya National Bureau of Statistics, World Health Organization. Kenya STEPwise Survey for Non Communicable Diseases Risk Factors 2015 Report 2015. Available from: http://www.who.int/chp/steps/Kenya_2015_STEPS_Report.pdf?ua=1. Accessed 4 Jan 2017.

[32] Krotkiewski M, Björntorp P, Sjöström L, Smith U (1983). Impact of obesity on metabolism in men and women. Importance of regional adipose tissue distribution. J Clin Invest.

[33] Kanter R, Caballero B (2012). Global gender disparities in obesity: a review. Adv Nutr.

[34] Pasco JA, Holloway KL, Dobbins AG, Kotowicz MA, Williams LJ, Brennan SL (2014). Body mass index and measures of body fat for defining obesity and underweight: a cross-sectional, population-based study. BMC Obesity.

[35] Derby CA, Zilber S, Brambilla D, Morales KH, McKinlay JB (2006). Body mass index, waist circumference and waist to hip ratio and change in sex steroid hormones: the Massachusetts male ageing study. Clin Endocrinol.

[36] Lasky D, Becerra E, Boto W, Otim M, Ntambi J (2002). Obesity and gender differences in the risk of type 2 diabetes mellitus in Uganda. Nutr.

[37] Lassek WD, Gaulin SJ (2006). Changes in body fat distribution in relation to parity in American women: a covert form of maternal depletion. Am J Phys Anthropol.

[38] Toth M, Tchernof A, Sites C, Poehlman E (2000). Effect of menopausal status on body composition and abdominal fat distribution. Int J Obes.

[39] Sobal J, Rauschenbach BS, Frongillo EA (1992). Marital status, fatness and obesity. Soc Sci Med.

[40] Evers S (1987). Economic and social factors associated with obesity in adult Canadians. Nutr Res.

[41] Amini M, Rezvanian H, Gouya M-M, Delavari A, Alikhani S, Mahdavi A (2008). Association of body mass index and abdominal obesity with marital status in adults. Arch Iran Med.

[42] Reeder BA, Senthilselvan A, Despres J, Angel A, Liu L, Wang H (1997). The association of cardiovascular disease risk factors with abdominal obesity in Canada. Canadian heart health surveys research group. Can Med Assoc J.

[43] Niskanen L, Laaksonen DE, Nyyssönen K, Punnonen K, Valkonen V-P, Fuentes R (2004). Inflammation, abdominal obesity, and smoking as predictors of hypertension. Hypertension.

[44] Larsson B, Svärdsudd K, Welin L, Wilhelmsen L, Björntorp P, Tibblin G (1984). Abdominal adipose tissue distribution, obesity, and risk of cardiovascular disease and death: 13 year follow up of participants in the study of men born in 1913. Br Med J (Clin Res Ed).

[45] Maher D, Waswa L, Baisley K, Karabarinde A, Unwin N, Grosskurth H (2011). Distribution of hyperglycaemia and related cardiovascular disease risk factors in low-income countries: a cross-sectional population-based survey in rural Uganda. Int J Epidemiol.

[46] Murphy GA, Asiki G, Ekoru K, Nsubuga RN, Nakiyingi-Miiro J, Young EH (2013). Sociodemographic distribution of non-communicable disease risk factors in rural Uganda: a cross-sectional study. Int J Epidemiol.

[47] Davey SG, Hart C, Hole D, MacKinnon P, Gillis C, Watt G (1998). Education and occupational social class: which is the more important indicator of mortality risk?. J Epidemiol Community Health.

[48] Okosun IS, Cooper RS, Rotimi CN, Osotimehin B, Forrester T (1998). Association of waist circumference with risk of hypertension and type 2 diabetes in Nigerians, Jamaicans, and African-Americans. Diabetes Care.

[49] Okosun IS, Liao Y, Rotimi CN, Choi S, Cooper RS (2000). Predictive values of waist circumference for dyslipidemia, type 2 diabetes and hypertension in overweight white, black, and Hispanic American adults. J Clin Epidemiol.

